# Do patients with celiac disease patients differ from those with concurrent celiac disease with type 1 diabetes mellitus?

**DOI:** 10.1186/1687-9856-2013-S1-O30

**Published:** 2013-10-03

**Authors:** Ashu Rastogi, Sanjay Kumar, Bhadada Rakesh Kochhar

**Affiliations:** 1 Senior Resident, Deptt. Of Endocrinology, PGIMER, Chandigarh, India; 2 Associate Professor, Deptt. Of Endocrinology, PGIMER, Chandigarh, India; 3 Professor, Deptt. Of Gastroenterology, PGIMER, Chandigarh, India

## Background and objectives

Celiac disease (CD) and type 1 diabetes mellitus (T1DM) share common genetic loci. Patients with T1DM developing CD may remain asymptomatic and some of the common symptoms of CD may be considered as part of the chronic complications of diabetes like gastrointestinal intolerance, diarrhea, nocturnal diarrhea, alternate diarrhea and constipation.

There are no reports on comparison of presenting features of patients with CD and those with CD and T1DM together. The present study was planned to compare clinical, biochemical and hormonal profile of patients of CD and CD with T1DM.

## Patients and methods

Consecutive CD patients with and without T1DM ≤ 20 years seen by us were evaluated clinically and underwent thyroid, gonadal function tests (where applicable) and serum cortisol, besides routine hemogram and biochemical tests. Patients were subjected to screening for CD by anti tissue transglutaminase antibodies (tTGAb) and those positive were subjected to endoscopy of descending part of the duodenum, and 3 biopsies were taken. Histological interpretation was done by an experienced pathologist and recorded as per modified Marsh classification [1]. Patients were diagnosed to have CD as per modified ESPGHAN criteria [2] and T1DM as per ADA position statement [3]. All patients received gluten free diet (GFD) and / or diabetic diet. Patients with CD alone (group A) were compared with those having CD with T1DM (Group B).

## Results

109 patients (57 males) with mean age of 14.9±2.9 year fulfilled the eligibility criteria. 23 (21.1%) had T1DM and CD while CD alone was present in 86 subjects. The age at diagnosis of CD was younger (11.5±4.6 vs 13.8 ±3.4 yr; p<0.05) and the lag period between symptoms and diagnosis of CD was shorter (20.2 ± 31.8 vs 56.1 ± 42.4 months; p<0.05) in those with Group B. Detailed clinical features of patients in two groups are given in Table 1 and laboratory parameters are enumerated in Table 2. Comparing the clinical features in between groups, short stature (87% versus 40.9%, p < 0.0001), anemia (80.9% versus 45%, p<0.001) and delayed puberty (61.9% versus 29.4%, p<0.014) were significantly more common in the Group A. However, diarrhea, constipation and weight loss were comparable in both the groups.

**Table 1:** Symptoms in patients with celiac disease alone and celiac disease with type 1 diabetes mellitus

S.No	Clinical Features	All Patients (N= 109)	CD Alone (Group A, N=86)	CD+T1DM (Group B, N=23)	P value (Group A&B))
1.	Short stature	77.1%	87%	40.9%	.000

2.	Diarrhoea	56.9%	40.7%	50%	.431

3.	Constipation	4.6%	3.5%	9.5%	.246

4.	Anemia	70.6%	80.9%	45%	.001

5.	Delayed Puberty	52.3%	61.9%	29.4%	.014

6.	Weight loss	55%	61.9%	45%	.168

7.	Hypothyroidism	8.2%	8.1%	5%	.788

8.	Rickets	3.7%	3.5%	4.3%	.096

9.	Goitre	19.3%	20.9%	13%	.704

10.	Hypoadrenalism	0.9%	1.2%	0%	.246

**Table 2:** Laboratory parameters of patients with celiac disease alone and celiac disease with type 1 diabetes mellitus.

S.No	Biochemical Parameters	All Patients (N= 109)	CD Alone (Group A, N=86)	CD+T1DM (Group B, N=23)	P value (Group A&B))
1.	Serum IgA tTG (mIU/L)	123.2±110.9	118.3±112.9	139.5±111.1	.433

2.	Hemoglobin (gm/dl)	8.6±2.6	8.3±2.4	10.1±3.2	.018

3.	T3	1.3±0.43	1.3±0.4	1.4±0.6	

4.	T4	8.1±2.6	8.1±2.7	7.7±3.1	

5.	TSH(mIU/L)	9.7±34.5	7.1±21.7	22.4±69.5	.146

6.	LH(mIU/L)	2.5±2.6	2.6±2.8	1.9±1.8	.596

7.	Calcium (mg/dl)	9.35±1.87	8.9±0.9	9.4±1.9	

8.	Phosphate (mg/dl)	4.7±1.0	4.7±1.0	4.4±0.83	

9.	ALP(IU/L)	118.0±108.4	110.2±106.4	116.6±129.5	

10.	Albumin (gm/dl)	4.0±0.6	4.05±0.7	4.3±0.5	

11.	SGOT	39.2±26.5	32.8±16.8	39.7±42.1	

12.	SGPT	36.3±31.2	28.6±15.6	34.2±58.0	

## Conclusions

Present study suggests that patients with celiac disease alone are more symptomatic and associated with more biochemical abnormalities compared to those with celiac disease and T1DM. It will be worthwhile to plan a prospective study with long duration of follow up and more number of patients in both groups to further validate the results.
